# Unmasking of molecular players: proteomic profiling of vitreous humor in pathologic myopia

**DOI:** 10.1186/s12886-024-03584-6

**Published:** 2024-08-19

**Authors:** Yue Wen, Chi Ren, Li Zhu, Lvzhen Huang, Huijun Qi, Wenzhen Yu, Kai Wang, Mingwei Zhao, Qiong Xu

**Affiliations:** 1 Department of Ophthalmology, Beijing Key Laboratory of Diagnosis and Therapy of Retinal and Choroid Diseases, Peking University People’s Hospital, Beijing, China; 2 College of Optometry, Peking University People’s Hospital, Beijing, China; 3 Eye Disease and Optometry Institute, Beijing, China; 4 Peking University Health Science Center, No. 11 South Avenue of XiZhiMen, Xi Cheng District, 100044 Beijing, China

**Keywords:** Pathologic myopia (PM), Proteomics, Chorioretinal cell apoptosis, Scleral extracellular matrix (ECM) synthesis, Scleral remodeling

## Abstract

**Background:**

This study aimed to identify the differentially expressed proteins in the vitreous humor (VH) of eyes with and without pathologic myopia (PM), providing insights into the molecular pathogenesis.

**Methods:**

A cross-sectional, observational study was conducted. VH samples were collected from patients undergoing vitrectomy for idiopathic epiretinal membrane (ERM), macular hole (MH), or myopic retinoschisis (MRS). Label-free quantitative proteomic analysis identified differential protein expression, with validation using ELISA.

**Results:**

The proteomic profiling revealed significantly higher expressions of tubulin alpha 1a (TUBA1A) and eukaryotic translation elongation factor 1 alpha 1 (EEF1A1) in PM groups (MH-PM, MRS-PM) compared to controls (MH, ERM). Conversely, xylosyltransferase 1 (XYLT1), versican core protein (VCAN), and testican-2 (SPOCK2) expressions were lower in PM. ELISA validation confirmed these findings.

**Conclusions:**

Our study provides novel insights into the molecular mechanisms of PM. The differentially expressed proteins EEF1A1, TUBA1A, XYLT1, VCAN, and SPOCK2 may play crucial roles in chorioretinal cell apoptosis, scleral extracellular matrix (ECM) synthesis, and scleral remodeling in PM. These proteins represent potential new targets for therapeutic intervention in PM, highlighting the importance of further investigations to elucidate their functions and underlying mechanisms in disease pathogenesis.

**Supplementary Information:**

The online version contains supplementary material available at 10.1186/s12886-024-03584-6.

## Background

Pathologic myopia (PM) is a leading cause of visual impairment worldwide. The International Myopia Institute (IMI) defines PM as excessive axial elongation due to myopia that can result in loss of best-corrected visual acuity as well as structural abnormalities in the posterior segment of the eye, such as posterior staphyloma, myopic maculopathy, and high myopia-associated optic neuropathy [1]. PM is the major cause of blindness and impaired vision in Chinese people older than 40 years [2, 3]. Furthermore, according to a global study, by 2050, there will be 938 million people with high myopia (9.8% of the world population), approximately half of whom will develop PM [4]. Consequently, research into the mechanism of pathologic myopia development is essential.

Myopia is known to be caused by a combination of environmental factors and genetics [5]. The most prominent pathological signs of myopia are scleral remodeling and severe axial elongation, which cause retinal thinning and even retinal detachment. The chorioretinal atrophic change is most commonly observed in pathologic myopic eyes. Chorioretinal atrophy worsens with increased myopic maculopathy grading [3], for which more research is needed to determine the underlying mechanism. Signaling pathways associated with inflammation, hypoxia, and apoptosis are essential for the development and occurrence of myopia. With regard to molecular mechanisms and signaling pathways, the pathogenesis of myopia may begin in the retina, progress through the choroid, and eventually reach the sclera, where it causes scleral remodeling [6–11]. Scleral remodeling is ensured by an imbalance between the synthesis and degradation of the scleral extracellular matrix (ECM), and many molecules are involved in this process [12].

Despite significant efforts to identify genetic risk factors for myopia, the genetic background, associated signaling pathways and therapy for PM have not been fully illustrated [3]. Proteomic analysis has been applied in numerous ocular disorders, including glaucoma, cataracts, and diabetic retinopathy, and aims to identify proteomic peptide biomarkers of disease for diagnosis and explanation of the mechanism for delivering innovative therapeutics [13]. Several ocular fluids, including aqueous humor, tears, and vitreous humor (VH), have been applied in previous proteomic research [14]. The VH is close to the retina and may reflect the retina’s environment. As a result, we employed a label-free quantitative proteomic analysis to look for differentially expressed proteins in VH samples from PM patients and controls.

## Methods

### Study design and patients

This was a cross-sectional, observational study of consecutive patients who underwent vitrectomy for myopic retinoschisis (MRS), idiopathic epiretinal membrane (ERM), or macular hole (MH) between December 2022 and May 2023. Patients underwent comprehensive preoperative eye examinations, including visual acuity, intraocular pressure, spherical equivalent refractive error (SER) evaluation, axial length (AL) (IOLMaster 700, ZEISS, Germany), and optical coherence tomography (RTVue100-2, Optovence, USA). Clinical information such as age, sex, surgical history, and systemic disorders was also collected.

For the inclusion in the PM group, patients were selected based on the presence of both SER <-6.0 D and AL > 26 mm, coupled with a fundus examination indicating pathological myopic changes. Conversely, patients with high myopia but lacking pathological fundus findings were excluded from this group. For the control group, patients undergoing vitrectomy due to ERM or MH, with SER ≥ -6.0 D and AL<26 mm, were enrolled. Pathological fundus characteristics comprising posterior staphyloma, diffuse or patchy chorioretinal atrophy, macular atrophy, lacquer cracks, myopic choroidal neovascularization, and Fuchs spot were assessed. Additionally, patients with a history of ocular surgery, intravitreal injection, autoimmune retinal diseases, or medication use potentially affecting the VH were excluded from both groups.

### VH sample collection

Prior to standard three-port pars plana vitrectomy (PPV; Constellation; Alcon Instruments, USA), VH samples (500–1000 µL) were obtained. A closed infusion tube was placed after a 25-gauge trocar was implanted into the inferior temporal sclera. After opening the vitrector aspiration line’s stopcock, a sterile five-millilitre syringe was inserted. A superior temporal port was used to introduce the vitrector. A total of 500–1000 µL of dry vitreous fluid was aspirated into the syringe using active cutting and syringe suction. The aspiration line was then closed, and infusion was started to stabilize the intraocular pressure. The samples were then put into sterile 1.5-mL microcentrifuge tubes. After snap-freezing, all the VH samples were kept at -80 °C until analysis.

### Sample preparation and liquid chromatography‒mass spectrometry (LC‒MS/MS)

After centrifugation at 12,000 × g for ten minutes at 4 °C, the cellular debris from the VH sample was separated. A bicinchoninic acid (BCA) protein assay kit (Beyotime Biotechnology, Shanghai, China) was used to measure the protein concentration after the supernatant was transferred to a fresh centrifuge tube following the manufacturer’s instructions. The protein mixture was reduced with 5 mmol/L dithiothreitol for 30 min at 56 °C and alkylated with 11 mmol/L iodoacetamide for 15 min at room temperature in darkness to facilitate digestion. Next, 100 mmol/L TEAB was added to urea concentrations less than 2 mol/L to dilute the protein sample. Trypsin was added at a ratio of 1:50 to the protein mass for the first overnight digestion and 1:100 to the protein mass for the second four-hour digestion. Finally, a C18 SPE column desalted the peptides.

### LC‒MS/MS data acquisition (4D mass spectrometer)

After dissolving the tryptic peptides in solvent A (0.1% formic acid, 2% acetonitrile/in water), the peptides were subsequently loaded onto a homemade reversed-phase analytical column (25 cm length, 75/100 µm i.d.). With a constant flow rate of 450 nL/min on a nanoElute UHPLC system (Bruker Daltonics), peptides were separated using a gradient from 6 to 24% solvent B (0.1% formic acid in acetonitrile) over 70 min, 24–35% in 14 min, increasing to 80% in three minutes, and holding at 80% for the final three minutes.

A timsTOF Pro mass spectrometer (Bruker Daltonics) was used to analyze the peptides after they underwent capillary source analysis. The electrospray voltage used was 1.60 kV. With an MS/MS scan range of 100 to 1,700 m/z, precursors and fragments were examined via the TOF detector. In parallel accumulation serial fragmentation (PASEF) mode, the timsTOF Pro was used. Ten PASEF-MS/MS scans were obtained each cycle, and precursors with charge states ranging from zero to five were chosen for fragmentation. The dynamic exclusion time was set to 30 s.

### Database search

To process all the raw data, the Proteome Discoverer search engine (v2.4.1.15) was used. Tandem mass spectra were compared to those of the Homo_sapiens_9606_SP_20220107.fasta database (20,376 entries), which was concatenated with the reverse decoy database. Trypsin/P was specified as the cleavage enzyme, allowing up to two missing cleavages. The mass tolerance for precursor ions was set to 10 ppm, and the mass tolerance for fragment ions was set to 0.02 Da. While acetylation on the protein N-terminal, oxidation on methionine, met-loss on methionine, and met-loss plus acetylation on methionine were selected as variable modifications, carbamidomethyl on cysteine was classified as a fixed modification. For peptide, protein, and peptide-spectrum match identification, the false discovery rate was set to 1%.

### Validation using an enzyme-linked immunosorbent assay (ELISA)

The concentrations of selected differentially expressed proteins in the VH were measured using ELISA kits (RUIFAN Biotech, Shanghai, China; Fine Biotech, Wuhan, China) following the manufacturer’s instructions. The samples were placed into the wells of a 96-well plate that had been coated with a monoclonal antibody. The plate was then washed, and another antibody tagged with an enzyme was added after the plate had been incubated for 30 min. Substrates A and B were added to the plate after it had been further incubated and cleaned. Using a microplate reader (SpectraMax 190, Molecular Devices, USA), the optical density was measured at 450 nm until color development ended. A standard curve was generated by measuring the optical densities of serially diluted protein solutions of known concentrations. The concentrations of EEF1A1, TUBA1A, XYLT1, VCAN, and SPOCK2 in the VH were calculated according to the standard curve.

### Statistical analysis

The ratio of the relative quantitative mean values of each differentially expressed protein in the PM and control samples was used as the fold change (FC). For example, calculate the FC in protein between the PM and control group. The formula is calculated as follows:

$$\begin{array}{l}F{C_{PM/control,k}} = Mean({R_{ik}},i \in PM)/\\Mean({R_{ik}},i \in control)\end{array}$$

where *R* indicates the relative quantitative value of the protein, *i* indicates sample and *k* indicates the protein. The significance of differences in relative quantitative values of each protein between two groups was analyzed using Student’s *t* test, with values undergoing log2 logarithmic transformation to conform to the normal distribution before the *t* test. The formula is calculated as follows:

$${P_k} = T.test(Log2({R_{ik}},i \in PM),Log2({R_{ik}},i \in control))$$

When the *P* value < 0.05 by the above difference analysis, the change in differential expression of more than 1.5 was used as the threshold of change for significant up-regulation, and less than 1/1.5 was used as the threshold of change for significant down-regulation. Differences between the PM and control groups were analyzed using Student’s *t* test and chi-square test, which included age, sex, SER, AL, and concentration of differentially expressed proteins. All the statistical analyses were performed using IBM SPSS Statistics 24. A two-tailed *P* value < 0.05 was considered to indicate statistical significance.

## Results

### Patient characteristics

A total of ten VH samples were collected from ten patients: three from MH-PM patients, two from MRS-PM patients, three from MH patients and two from ERM patients. The characteristics of the PM patients and control patients are shown in Table 1 and Supplementary Table 1. Age and sex distributions were not significantly different between the two groups, while patient SER, patient AL were significantly different between the two groups.

**Table 1:** Patient characteristics

Characteristics	PM group	Control group	*P* ^*^
Age (year)	59.2 ± 16.9	67.0 ± 7.2	0.371
Gender (male/female)	1/4	2/3	0.545
SER (D)	-17.03 ± 6.86	-0.73 ± 2.12	0.001
AL (mm)	30.38 ± 2.21	23.18 ± 1.03	< 0.001
Posterior staphyloma	2	0	-
Chorioretinal atrophy	5	0	-

The values represent the number of patients or the mean and range. ^*^, Student’s *t* test (age, SER and AL) or chi-square test (sex). PM, pathologic myopia; SER, spherical equivalent refractive error; D, dioptres; AL, axial length

### Differentially expressed proteins between the PM and control groups

**Fig. 1:**
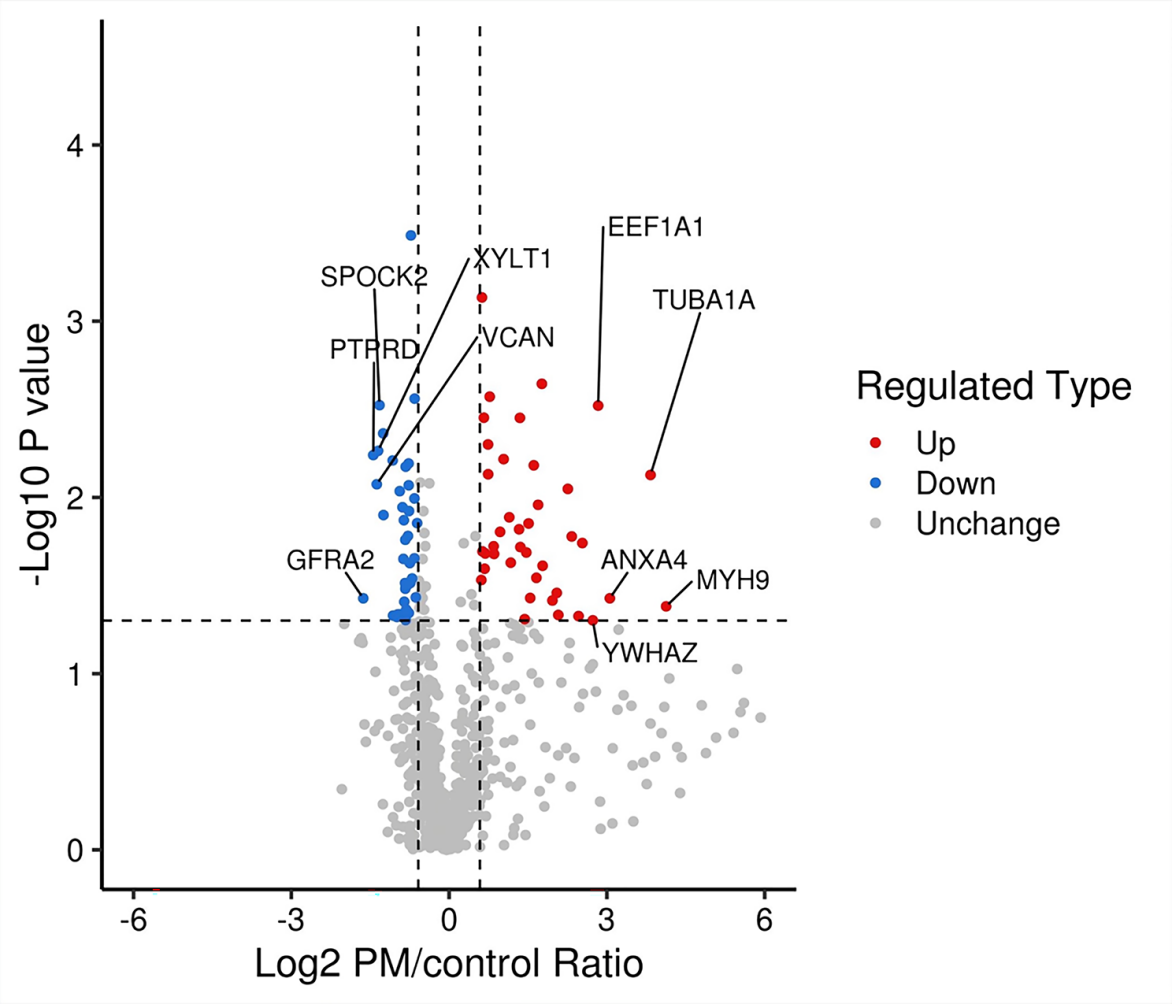
Volcano plots represent the differentially expressed proteins. The relative quantitative mean values of each differentially expressed protein in the PM and control samples were used to calculate the PM/control ratio. The horizontal axis shows the fold changes in the expression of the genes in the two groups (PM/control ratio) after log2 transformation. The vertical axis is the *P* value converted by -Log10. Thirty-nine proteins were upregulated in the PM samples (red dots), 38 proteins were downregulated (blue dots), and the gray dots indicate proteins with no significant difference in expression. The top 5 upregulated and downregulated proteins are also marked. EEF1A1, eukaryotic translation elongation factor 1 alpha 1; TUBA1A, tubulin alpha 1a; ANXA4: annexin A4; MYH9, myosin-9; YWHAZ, 14-3-3 protein zeta/delta; GFRA2: GDNF family receptor alpha-2; SPOCK2, testican-2; PTPRD, receptor-type tyrosine-protein phosphatase delta; XYLT1, xylosyltransferase 1; VCAN, versican core protein (VCAN)

**Fig. 2:**
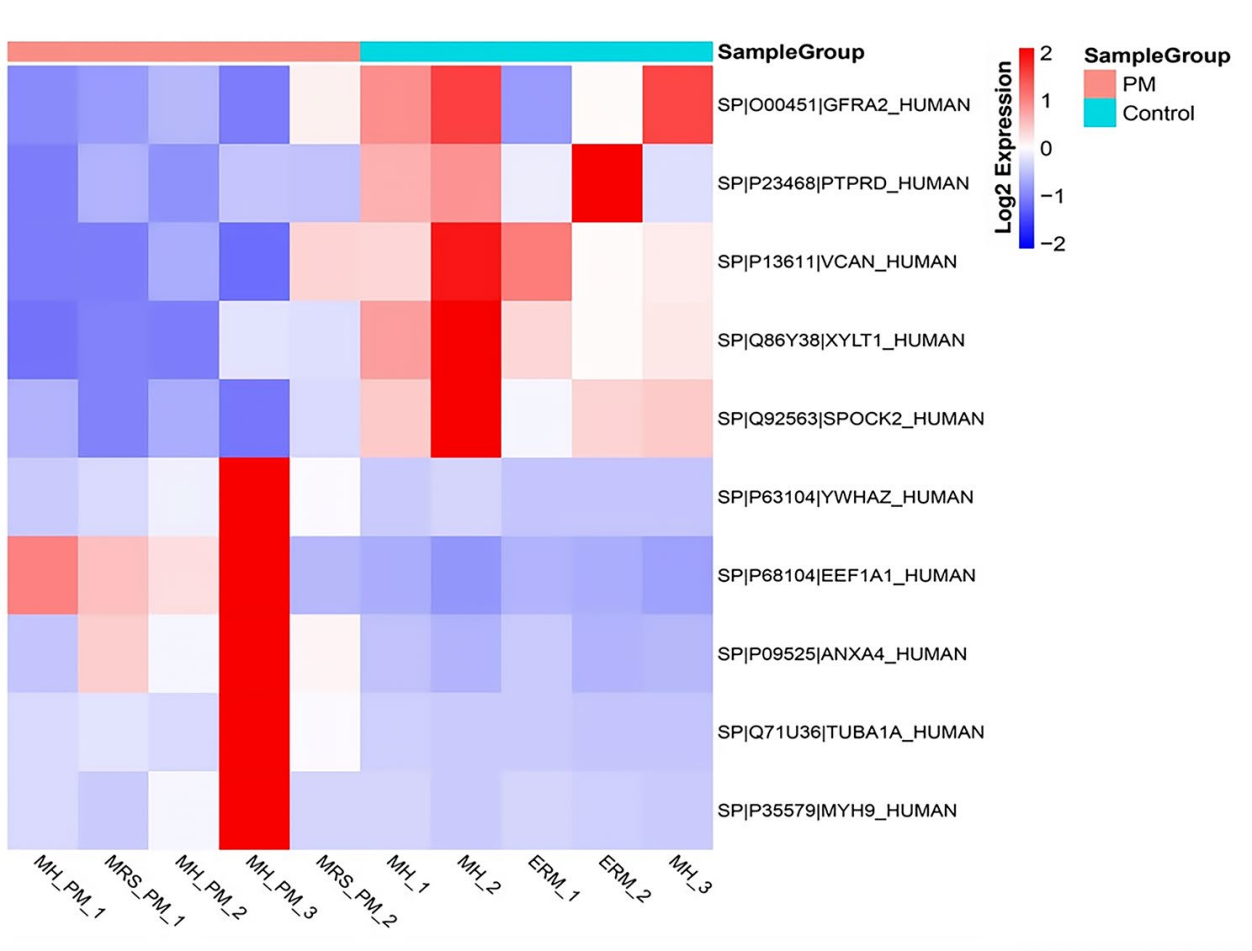
The heatmap shows the relative expression levels of proteins undergoing log2 logarithmic transformation in different samples. The sample names are listed at the bottom, while each row represents a differentially expressed protein. (red = up-regulation, blue = downregulation). VH, vitreous humor; PM, pathologic myopia; MH, macular hole; MRS, myopic retinoschisis; ERM, epiretinal membrane

In total, 77 proteins were found to be differentially expressed in the VH samples between the PM and control groups (detailed in Supplementary Tables 2 and 3). Thirty-nine proteins were upregulated in the PM group, while 38 proteins were downregulated. Volcano plots were constructed to determine the fold change in protein expression (Fig. 1). The top 5 upregulated and downregulated proteins were also marked. The expression levels of eukaryotic translation elongation factor 1 alpha 1 (EEF1A1), tubulin alpha 1a (TUBA1A), annexin A4 (ANXA4), myosin-9 (MYH9) and 14-3-3 protein zeta/delta (YWHAZ) were significantly greater than those in the control group, while the expression levels of the GDNF family receptor alpha-2 (GFRA2), testican-2 (SPOCK2), receptor-type tyrosine-protein phosphatase delta (PTPRD), xylosyltransferase 1 (XYLT1) and versican core protein (VCAN) were significantly lower than those in the control group. A heatmap was constructed for the ten differentially expressed proteins, as shown in Fig. 2. It showed the relative expression levels of ten differentially expressed proteins in different samples, with values undergoing log2 logarithmic transformation.

### Validation of alterations in protein expression using ELISA

**Fig. 3:**
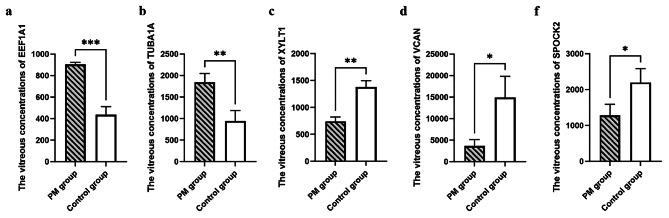
ELISA of three proteins showing different expression (pg/mL). (**a**) The vitreous concentrations of EEF1A1. (**b**) The vitreous concentrations of TUBA1A. (**c**) The vitreous concentrations of XYLT1. (**d**) The vitreous concentrations of VCAN. (**f**) The vitreous concentrations of SPOCK2. PM, pathologic myopia; EEF1A1, translation elongation factor 1 alpha 1; TUBA1A, tubulin alpha 1a; XYLT1, xylosyltransferase 1; VCAN, versican core protein (VCAN); SPOCK2, testican-2

ELISA was used to examine the levels of three VH proteins (EEF1A1, TUBA1A, XYLT1, VCAN and SPOCK2), which were selected with large absolute values of -log10 (*P* value) and log2 (PM/control ratio) from Fig. 1, to confirm the expression of differentially expressed proteins demonstrated by the label-free quantitative proteomic analysis. The vitreous EEF1A1, TUBA1A, XYLT1, VCAN, and SPOCK2 concentrations were determined using three random VH samples from each group. Fig. 3 displays the ELISA results (detailed in Supplementary Table 4). EEF1A1 and TUBA1A concentrations in the VH were greater in PM patients (903.97 ± 19.36 pg/mL; 1838.43 ± 208.05 pg/mL) than in controls (436.22 ± 75.16 pg/mL, *P* < 0.001; 942.60 ± 245.05 pg/mL, *P* = 0.008), while XYLT1, VCAN, and SPOCK2 concentrations were lower in PM patients (739.80 ± 82.62 pg/mL; 3641.39 ± 1465.40 pg/mL; 1279.15 ± 313.07 pg/mL) than in controls (1376.78 ± 120.55 pg/mL, *P* = 0.002; 14930.39 ± 4889.78 pg/mL, *P* = 0.0186; 2193.72 ± 390.36 pg/mL, *P* = 0.0340).

## Discussion

PM, a leading cause of blindness in adults, is associated with scleral thinning, choroidal atrophy, and retinal detachment [15]. Despite its significance, the underlying mechanisms of PM remain elusive. Limited availability of animal models and human retinal samples has propelled the use of proteomics to study differentially expressed proteins in the vitreous humor (VH) of PM patients [16]. Our study profiled VH proteins from PM and non-PM patients, identifying key differentially expressed proteins: EEF1A1 and TUBA1A were upregulated, while XYLT1, VCAN, and SPOCK2 were downregulated, confirmed by ELISA.

A previous study revealed the downregulation of prostaglandin-H2 D-isomerase (PGDS), glutathione peroxidase 3 (GPX3) and nuclear factor erythroid 2-related factor 2 (NRF-2) in patients with ALs greater than 29.0 mm. PGDS, which catalyzes the production of prostaglandin D2, is involved in transporting lipophilic substances in the interphotoreceptor matrix, influencing immunological and inflammatory responses, and causing apoptosis and neuroprotection. To protect cells from oxidative damage, GPX3 catalyzes the reduction of organic hydroperoxides and hydrogen peroxide. NRF-2, a significant antioxidative protein, is implicated in the etiology of many diseases and serves as a cellular autonomic defense system. As a result, researchers have hypothesized that an oxidation/antioxidation imbalance could be a key mechanism underpinning the pathogenesis of PM-related retinopathy [16]. According to our research, patients with pathologic myopia had downregulated expression of XYLT1, VCAN, and SPOCK2 and upregulated expression of TUBA1A and EEF1A1.

**Fig. 4:**
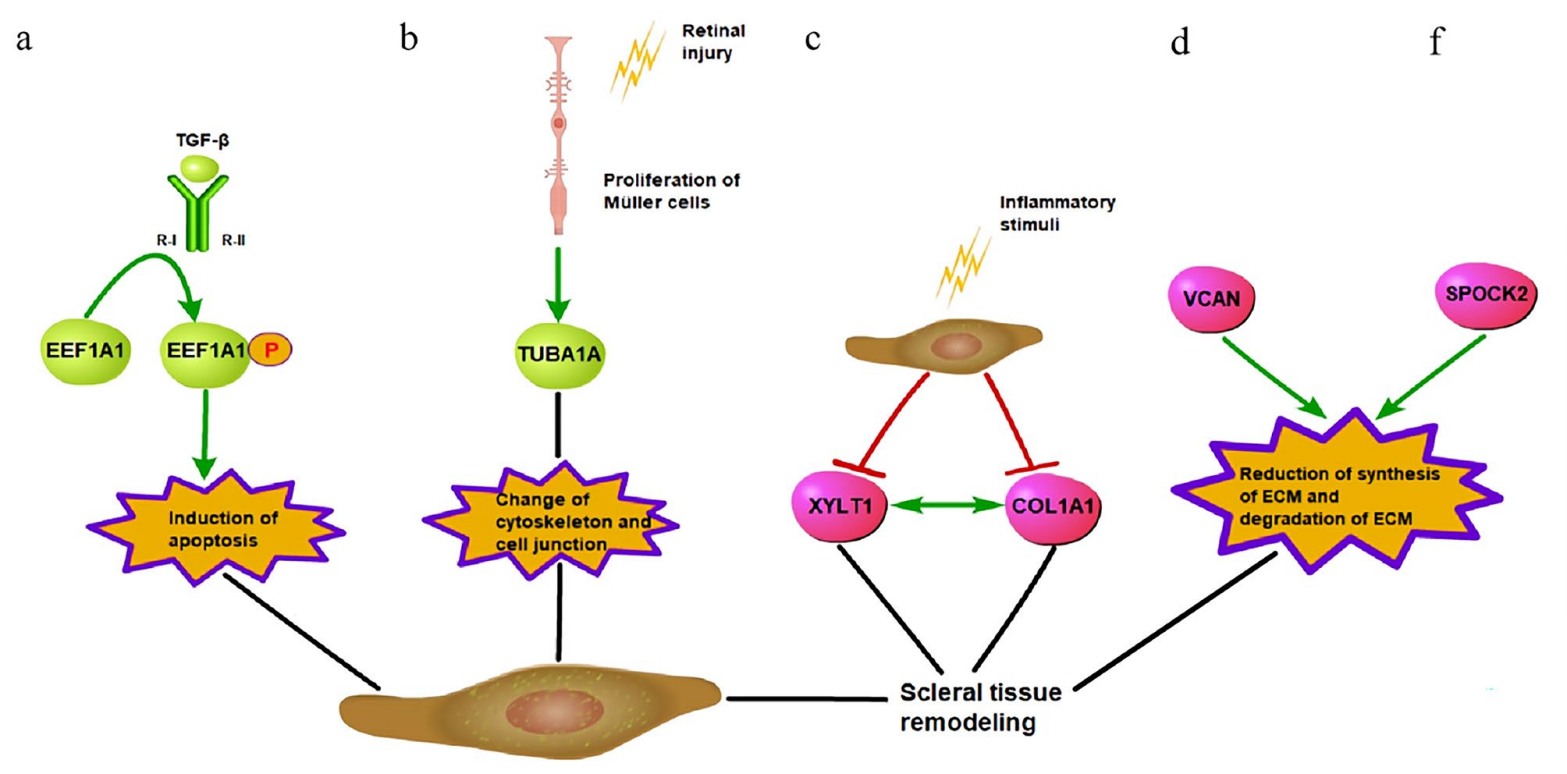
Potential signaling pathways related to scleral remodeling in patients with pathologic myopia. (**a**) Transforming growth factor β (TGF-β) phosphorylates EEF1A1 by activating the transforming growth factor β type I receptor (TβR-I), which induces chorioretinal cell apoptosis. (**b**) Retinal injury stimulates proliferation of Müller cells and overexpression of TUBA1A, which interferes with the cytoskeleton and cell junctions. (**c**) An inflammatory environment downregulates expression of XYLT1 and COL1A1, then reduces scleral extracellular matrix synthesis. (**d**) Downregulation of VCAN reduces the synthesis of ECM in scleral fibroblasts in PM. (**f**) Downregulation of SPOCK2 in patients with PM may degrade the scleral ECM. Green color represents upregulation of gene expression level, red color represents downregulation of expression level. All of these signaling pathways may lead to scleral tissue remodeling. COL1A1, collagen type I alpha 1

EEF1A1 is not only a translation factor but is also a pleiotropic protein found in human cancers such as breast cancer, ovarian cancer, and lung cancer [17]. EEF1A1 is the most prevalent gene in the retinal pigment epithelium (RPE) and the second most abundant gene in the iris in humans, although it is also strongly expressed in other tissues [18]. Previous research has shown that EEF1A1 controls the cytoskeleton, has chaperone-like activity, is involved in the remodeling of microtubules and filamentous actin, and may mediate cytoskeletal alterations involved in lipotoxic cell death [19, 20]. The role of EEF1A1 in apoptosis has been controversial. EEF1A1 seems to promote apoptosis, while a previous study showed that EEF1A1 only exhibits antiapoptotic effects when the proapoptotic protein Bax is present [21]. Our prior research revealed that cell apoptosis is an important contributor to the onset and progression of myopia. The proapoptotic proteins Bax, Caspase-3, and Caspase-8 were upregulated in the retinas of chicks undergoing form deprivation, whereas the antiapoptotic protein Bcl-2 was downregulated, indicating that retinal cell apoptosis may be an important mechanism in the development of myopia. This study compared changes in protein expression in the VH between patients with PM and controls using proteomics. EEF1A1 exhibited the greatest difference in expression among the proteins with significant differential expression. Interestingly, Lin KW et al [22] proposed that in breast cancer, transforming growth factor β (TGF-β) phosphorylates EEF1A1 by activating the transforming growth factor β type I receptor (TβR-I), which inhibits cell proliferation. We wondered whether EEF1A1 plays a significant role in the pathogenesis of PM by participating in TGF-β-mediated chorioretinal cell apoptosis, as shown in Fig. 4. We eagerly anticipate conducting additional studies in the future to corroborate these findings.

The TUBA1A gene encodes tubulin alpha 1a, a protein with an important role in microtubule function and stability that is expressed in both the developing brain and retinal tissue. According to earlier research, overexpression of TUBA1A may disrupt the activities of the cytoskeleton and cell junctions, resulting in congenital cataracts, microphthalmia, and brain malformations [23–25]. Ramachandran R et al [26] discovered that TUBA1A is not expressed in the adult zebrafish retina but that retinal injury can stimulate TUBA1A expression in Müller cells that have reentered the cell cycle. Müller cells proliferate reactively in PM patients due to excessive axial elongation [27]. Although there have been no reports that TUBA1A plays a role in the development of myopia, we found a significant increase in TUBA1A expression in the VH of PM patients. We wondered if atrophic and damaged retinas in patients with PM cause overexpression of TUBA1A after reactive proliferation of Müller cells. Then it may interfere with the cytoskeleton and cell junctions, and eventually affects scleral remodeling and leads to the continuous progression of PM (Fig. 4), similar to what occurs in zebrafish with retinal damage. We cannot help but wonder whether the increase in TUBA1A expression will be used as an indicator protein for the development of PM in the future. It may help limit the progression of PM and become a new therapeutic target by blocking the expression of TUBA1A expression.

Human xylosyltransferase-I (XT-I), encoded by the gene XYLT1, is a type II transmembrane protein and a key enzyme in glycosaminoglycan (GAG) biosynthesis [28, 29]. GAG chains and a proteoglycan core protein make up proteoglycans, which are essential parts of the extracellular matrix (ECM) [29]. Scleral fibroblasts secrete a variety of proteins, including type I collagen and proteoglycans. Reduced synthesis of proteoglycans due to downregulated expression of XYLT1 may promote scleral remodeling, eventually leading to exophthalmos and blue sclera [30]. Scleral remodeling in myopic patients is also linked to the general loss of type I collagen and proteoglycans in the posterior pole of the sclera [8, 31]. Ye M et al [32] found the XYLT1 gene mutation in patients with high myopia using whole-exome sequencing. However, further research is still needed to determine the specific mechanism underlying the occurrence of the XYLT1 gene mutation in high myopia. For the first time, we found that patients with PM had significant downregulation of XYLT1 expression in the VH. In a previous study, Ly TD et al [29] reported that XYLT1 expression was significantly decreased when lipopolysaccharide (LPS) or adenosine triphosphate was used to simulate an inflammatory environment in human dermal fibroblasts in vitro. XYLT1 expression was downregulated in human proto-myofibroblasts subjected to acute senescence via hydrogen peroxide. The expression of collagen type I alpha 1 (COL1A1) was also shown to be significantly decreased [33]. Downregulation of XYLT1 expression causes a decrease in ECM synthesis and results in fibrous tissue remodeling. Prior research has demonstrated that chronic inflammation plays an important role in the progression of myopia. The inflammatory stimulant LPS can promote the development of myopia. Additionally, inflammatory factors have been found to be overexpressed in animal models of myopia [9]. Yoshimasa Ando et al. also confirmed that high myopia is associated with inflammation [34]. There still remained to be unknown that if the inflammatory environment in PM impacts scleral fibroblasts, which subsequently downregulate the expression of XYLT1. The downregulated expression of XYLT1 leads to the loss of proteoglycans and type I collagen, reducing the synthesis of ECM and ultimately resulting in scleral remodeling, as shown in Fig. 4. However, the specific underlying mechanism remains to be discovered.

Additionally, we detected downregulation of VCAN, SPOCK2 in vitreous humor of PM group. The versican core protein encoded by VCAN is large chondroitin sulfate proteoglycans of the ECM. The C-terminal G3 domain of versican binds other extracellular matrix molecules and forms a supramolecular structure that stores TGF-β and regulates its signaling [35]. Versican is also involved in ECM synthesis in scleral fibroblasts and is associated with myopia [36]. Downregulation of VCAN reduces the synthesis of ECM in scleral fibroblasts in PM, which ultimately leads to scleral remodeling and elongation of the axial length. SPOCK2 encodes the protein testican-2 that binds to glycosaminoglycans to form part of the ECM. Wang C et al. founded SPOCK2 could act as an upstream signaling molecule to regulate the activation of matrix metalloproteinase-2 (MMP-2), upregulation of SPOCK2 inhibited MMP-2 expression [37]. Downregulation of SPOCK2 in patients with PM may degrade the scleral ECM through activation of MMP-2 expression, ultimately leads to scleral remodeling. These signaling pathways in Fig. 4 are based on our and other investigators’ studies, and further experiments will be conducted to verify these pathways.

Unlike prior proteomic studies on PM, we did not include patients with retinal detachment when selecting patients with PM. We tried our best to reduce the impact of RPE exposure in individuals with retinal detachment by including only patients with PM-related macular holes and retinoschisis. Furthermore, while the sample size of this study was small, we uncovered previously unknown proteins connected to the PM. These proteins may be involved in scleral remodeling, retinal injury, and inflammatory pathways associated with PM.

## Conclusions

Our proteomic profiling of vitreous humor in PM revealed contrasting patterns of protein expression. Specifically, EEF1A1 and TUBA1A were upregulated, while XYLT1, VCAN, and SPOCK2 were downregulated. These findings indicate that these proteins may play pivotal roles in the pathogenesis of PM, including regulating chorioretinal cell apoptosis, cytoskeleton organization, and scleral extracellular matrix (ECM) synthesis. Our study sheds new light on the molecular mechanisms underlying PM and identifies potential therapeutic targets for this vision-threatening condition.

### Electronic supplementary material

Below is the link to the electronic supplementary material.

Supplementary Material 1

Supplementary Material 2

Supplementary Material 3

Supplementary Material 4

## Data Availability

Data is provided within the manuscript or supplementary information files.

## Abbreviations

VH: Vitreous humor
PM: Pathologic myopia
ERM: Epiretinal membrane
MH: Macular hole
MRS: Myopic retinoschisis
TUBA1A: Tubulin alpha 1a
EEF1A1: Eukaryotic translation elongation factor 1 alpha 1
XYLT1: Xylosyltransferase 1
VCAN: Versican core protein
SPOCK2: Testican-2
ECM: Extracellular matrix
ELISA: Enzyme-linked immunosorbent assay
SER: Spherical equivalent refractive error
AL: Axial length
LC-MS: Liquid chromatography‒mass spectrometry
